# Sex‐specific inbreeding depression: A meta‐analysis

**DOI:** 10.1111/ele.13961

**Published:** 2022-01-21

**Authors:** Regina Vega‐Trejo, Raïssa A. de Boer, John L. Fitzpatrick, Alexander Kotrschal

**Affiliations:** ^1^ 7675 Department of Zoology: Ethology Stockholm University Stockholm Sweden; ^2^ Department of Zoology Edward Grey Institute University of Oxford Oxford UK; ^3^ Behavioural Ecology Group Wageningen University & Research Wageningen The Netherlands

**Keywords:** heterogamous, homozygosity, incest, sexual conflict, sexual selection

## Abstract

Inbreeding depression, the reduced fitness of the offspring of related individuals, can affect males and females differently. Although a comprehensive theoretical framework describing the causes of sex‐specific inbreeding depression is lacking, empirical evidence suggests that often one sex tends to be more vulnerable than the other. However, the generality, direction, and degree of sex‐specific difference in inbreeding depression remains enigmatic as studies on this topic have reported conflicting results. Here, we conduct a meta‐analysis to test for sex‐specific differences in the magnitude of inbreeding depression. We synthetised 321 effect sizes of experimental studies across 47 species and found a small difference in inbreeding depression between the sexes: females suffered slightly higher inbreeding depression than males. Furthermore, a higher inbreeding coefficient was correlated with higher inbreeding depression. However, there was a large amount of heterogeneity that remained unexplained, even when considering different factors that could affect inbreeding between the sexes, such as sexual size dimorphism, heterogamety, the type of trait measured and whether animals were tested in a stressful environment. As such, we highlight the need to further explore inbreeding depression across different species to determine the occurrence and causes of sex differences to increase our understanding of the evolutionary consequences of sex‐specific inbreeding depression.

## Graphical abstract

Our meta‐analysis across experimental studies found evidence for general inbreeding depression for both males and females and, although the difference between the sexes was not substantial, females were slightly more sensitive to inbreeding depression than males. Our study highlights the need to further explore the effects of inbreeding depression due to intrinsic biological differences between males and females.

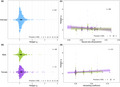

## INTRODUCTION

Inbreeding depression, the reduction in fitness in inbred compared to outbred individuals, is caused by the expression of deleterious recessive alleles and/or the consequent loss of heterozygous advantage (Charlesworth & Charlesworth, 1987). Both inbreeding and inbreeding depression are often detected in animal populations (Crnokrak & Roff, 1999; Keller & Waller, 2002). Empirical studies commonly demonstrate that males and females are affected differently by inbreeding depression (see Ebel & Phillips, 2016). Yet, whether inbreeding depression is generally stronger in one sex or the other remains unclear. Some studies show higher inbreeding depression in males (e.g. Mallet & Chippindale, 2011; Miller & Hedrick, 1993; Saccheri et al., 2005), while others report higher inbreeding depression in females (e.g. Ebel & Phillips, 2016; Fox et al., 2006; Sultanova et al., 2018). However, which sex is most vulnerable to inbreeding depression appears to vary based on the species, trait, and experimental/environmental conditions being examined (Ebel & Phillips, 2016). We therefore currently lack a clear understanding of the overall direction, causes and evolutionary consequences of sex‐specific inbreeding depression. Clarifying our understanding of sex‐specific inbreeding depression is crucial, as different responses to inbreeding between the sexes can have important implications for a wide range of ecological, evolutionary and conservation processes, including the evolution of mating systems, sex‐specific adaptive selection and the scope and potential for evolutionary rescue (Bonduriansky et al., 2008; Cassinello, 2005; Janicke et al., 2013).

Sex‐specific inbreeding depression can emerge if the strength of selection differs between males and females (Table 1). Males are generally expected to be under stronger sexual selection due to sex differences in gametic investment (i.e. anisogamy, Parker et al., 1972) and because males typically experience greater variance in reproductive success than females (Janicke et al., 2016; Wade, 1979). Consequently, males may be more sensitive to inbreeding depression than females, as deleterious alleles exposed from inbreeding should more directly impact male fitness (Grieshop et al., 2021; Rowe & Houle, 1997). Under this scenario, stronger sexual selection in males could lead to deleterious mutations being purged from the male genome more efficiently. Yet, sexual selection does not act solely on male‐specific alleles. Instead, sexually selected traits likely share a common genetic basis for traits that determine the overall health and vigour of individuals of both sexes (Cally et al., 2019; Ebel & Phillips, 2016; Mallet et al., 2011; Rowe & Houle, 1997). Males could still be more sensitive to inbreeding depression however, as stronger selective pressures lead to more directional dominance and selection is expected to remove dominant deleterious alleles quickly from a population (Wolak & Keller, 2014). Regardless of these genetic mechanisms, males are generally expected to be more sensitive to inbreeding depression than females, provided they are the sex that experiences stronger sexual selection (Table 1).

**TABLE 1:** Moderators used to evaluate sex‐specific inbreeding depression in a phylogenetically controlled meta‐analytical framework. A description of the expected effect of each moderator on the strength of inbreeding depression is provided. Predicted sex‐specific effects are provided for each moderator, specifying if the sex specific effects are predicted to be greater in males than females (M > F), greater in females than males (F > M), or equivalent between the sexes (M ≈ F). Model refers to the meta‐regressions tested in Table 2

Moderator	Link between moderator and inbreeding depression	Predicted sex‐specific effects	Model
Strength of [sexual] selection	Inbreeding depression is expected to be stronger with increasing strength of sexual selection (Ebel & Phillips, 2016; Grieshop et al., 2021; Mallet & Chippindale, 2011; Wolak & Keller, 2014)	M > F: Inbreeding depression is predicted to be higher in males than females as the strength of sexual selection is usually higher in males. As the strength of sexual selection increases (e.g. with increasing sexual size dimorphism), the magnitude of male‐biased sex‐specific inbreeding depression is predicted to increase	III VIII
Heterogamety	Homogametic sex is expected to be more affected by inbreeding depression (Carazo et al., 2016; Mallet & Chippindale, 2011; Sultanova et al., 2018)	F > M (XY system) and M > F (ZW system): Whether males or females are more or less sensitive to inbreeding depression is predicted to depend on which sex is the homogametic sex	IV IX
Inbreeding coefficient	Inbreeding depression is expected to be positively correlated with inbreeding coefficients (Charlesworth & Willis, 2009)	M ≈ F: There are no *a priori* predictions for sex‐specific inbreeding depression. However, if sex‐specific effects are present they may be more detectable at high inbreeding coefficients	V X
Type of trait	Traits closely related to fitness (e.g. life history traits) are expected to be more affected by inbreeding depression than traits less closely related to fitness (e.g. morphological traits; Chapman et al., 2009; DeRose & Roff, 1999)	M ≈ F: There are no *a priori* predictions for sex‐specific inbreeding depression. However, if sex‐specific effects are present they may be more detectable in traits closely related to fitness	VI XI
Environmental conditions	Inbreeding depression is expected to increase under more stressful environmental conditions (Armbruster & Reed, 2005; Fox & Reed, 2011)	M ≈ F: There are no *a priori* predictions for sex‐specific inbreeding depression. However, if sex‐specific effects are present they may be more detectable when environments are novel or stressful	VII XII

A major challenge when examining sex‐specific inbreeding depression is addressing the fact that both sexes share large portions of the genome (i.e. autosomal chromosomes). To address this challenge, sex‐specific inbreeding depression has been hypothesised to be influenced by the accumulation of deleterious recessive mutations in the sex chromosomes (Table 1; Sultanova et al., 2018; Trivers, 1985). In sex chromosome heteromorphic species, one sex carries a heterogamous set of sex chromosomes (the heterogametic sex, e.g. XY males in XY systems and ZW females in ZW systems) and the other a homogamous set (the homogametic sex, e.g. XX females in XY systems and ZZ males in ZW systems). Genes on the heterogametic sex (e.g. XY males/ZW females) cannot contribute to inbreeding depression, because deleterious recessive alleles on the sex chromosome cannot be masked by a second dominant allele and are thus expressed regardless of the degree of inbreeding (Agrawal, 2011; Sultanova et al., 2018). In contrast, in the homogametic sex (e.g. XX females/ZZ males) alleles on the sex chromosomes can contribute to inbreeding depression because the effects of recessive deleterious alleles may only be expressed after inbreeding (Brengdahl et al., 2018). Heteromorphic sex chromosomes can therefore lead to sex‐specific inbreeding depression simply because there are more genes available to contribute to inbreeding depression in the homogametic sex. Although this may not necessarily implicate a large number of genes contributing to inbreeding depression, their effects on fitness may be disproportional because sex chromosomes often constitute an arena for the resolution of sexual conflict over different trait optima between males and females (Ellegren & Parsch, 2007). Sex chromosomes are therefore likely to accumulate sexually antagonistic alleles (Rice, 1984; but see Fry, 2010). Alleles that only benefit the heterogametic sex will need to be recessive in order to persist because only then are they shielded from selection in the homogametic sex (Bonduriansky & Chenoweth, 2009; Rice, 1984). Inbreeding would expose such recessive sexually antagonistic alleles that benefit the heterogametic sex but harm the homogametic sex (Robinson et al., 2014). Taken together, the homogametic sex (XX females in XY systems and ZZ males in ZW systems) is expected to be more affected by inbreeding depression than the heterogametic sex (Table 1; Carazo et al., 2016; Ebel & Phillips, 2016; Mallet et al., 2011).

Detecting sex‐specific inbreeding depression (if present) likely depends on the conditions and traits being evaluated. For example, inbreeding depression is expected to be more deleterious when individuals mate with more closely related relatives (i.e. as inbreeding coefficients increase, Charlesworth & Willis, 2009). Inbreeding depression can also increase under novel or stressful environmental conditions due to genotype by environmental interactions (Armbruster & Reed, 2005; Bijlsma et al., 1999, 2000; Fox & Reed, 2011; Kristensen & Sorensen, 2005). Moreover, traits closely associated with fitness are more likely to be affected by inbreeding depression (DeRose & Roff, 1999), as fitness traits tend to display low additive genetic variation, but high directional dominance (Crnokrak & Roff, 1995; Saccheri et al., 2005). Traits directly correlated with individual fitness (i.e. life history traits such as survival and reproductive success) are therefore expected to be more sensitive to inbreeding depression than traits likely to be less closely related to individual fitness (e.g. morphological traits such as body size; Chapman et al., 2009; DeRose & Roff, 1999). Incorporating information on inbreeding coefficients and environmental stress, while examining a range of traits, is thus crucial when assessing sex‐specific inbreeding depression.

Yet predicting how different conditions and traits will influence sex‐specific inbreeding depression remains challenging. In the absence of sex‐specific differences in inbreeding depression caused by the explanations summarised above (i.e. sexual selection and heterogamety), any elevated costs of inbreeding depression that are associated with increasing inbreeding coefficients should be expressed similarly between the sexes (Table 1). It is also unclear whether males or females will be more sensitive to inbreeding depression under stressful environments (Table 1). The strength and direction of selection on males and females, and the subsequent costs associated with inbreeding depression, can align under stressful conditions making both sexes similarly sensitive to stress‐mediated inbreeding depression (Martinossi‐Allibert et al., 2017). However, as males and females often behave differently, sex differences in behaviour could lead to males and females varying in their ability to compensate for inbreeding depression (Charpentier et al., 2006). For example, stronger sexual selection on males is often associated with density dependent behavioural interactions, which reflects more intense intraspecific competition (i.e. a stressful environment; Yun & Agrawal, 2014), potentially leading to greater sensitivity to inbreeding depression in males. Finally, sex‐specific inbreeding depression among different traits could occur if fitness optima of trait expression differ between the sexes (Table 1; Bonduriansky & Chenoweth, 2009; Kokko & Jennions, 2014; Mallet & Chippindale, 2011). This is because sexually antagonistic selection may increase fitness when expressed in one sex but reduce it when expressed in the opposite sex (Bonduriansky & Chenoweth, 2009). How sexually antagonistic selection may drive sex‐specific inbreeding depression will depend on the concordance of mutational effects and the average degree of directional dominance between the sexes (Janicke et al., 2013; Mallet et al., 2011). Generating clear predictions about sex‐specific inbreeding depression among traits therefore requires the identification of specific traits where fitness optima differ between the sexes. Consequently, sex‐specific effects of inbreeding depression may be influenced by, and potentially be more readily detectable, depending on inbreeding coefficients, environmental stress, or the traits being examined, although in most cases it remains challenging to generate specific predictions.

Here, we consider two main processes that could explain sex‐specific inbreeding depression (differences in the strength of sexual selection, and the accumulation of deleterious mutations in the sex chromosomes through heterogametic vs. homogametic sex heterogamety) and highlight other factors that could affect our ability to detect sex‐specific patterns (Table 1). As it is currently unclear how prevalent sex‐specific inbreeding depression is and which sex is more affected by inbreeding depression (Table 1), we summarise the evidence for sex‐specific inbreeding depression across a range of traits and species in a phylogenetically controlled meta‐analysis. We specifically sought to answer the following questions and their associated predictions: (i) *Does inbreeding depression differ between the sexes?* Males are predicted to be more sensitive to inbreeding depression than females due to differences in the strength of sexual selection. (ii) *Is sexual size dimorphism associated with sex*‐*specific inbreeding depression?* A corollary of the prediction from question (i) is that the difference in inbreeding depression between the sexes will increase as the difference in the strength of sexual selection between the sexes increases. We therefore predict that differences in inbreeding depression between the sexes will increase with sexual body size dimorphism, a proxy for sexual selection that correlates with the opportunity for sexual selection (Janicke & Fromonteil, 2021). (iii) *Can heterogamety explain differences in inbreeding depression between the sexes?* The homogametic sex (XX females in XY systems and ZZ males in ZW systems) is predicted to be more affected by inbreeding depression than the heterogametic sex. (iv) *Are males and*/*or females differently affected depending on their inbreeding coefficient?* Inbreeding depression is predicted to be positively correlated with inbreeding coefficients. (v) *Are males and*/*or females more sensitive to inbreeding depression depending on the trait measured?* Traits more closely related to fitness (mating success, reproductive output and survival) are predicted to be more sensitive to inbreeding depression than traits less closely related to fitness (body size). (vi) *Are the sexes affected differently by inbreeding depression when exposed to environmental stress?* Inbreeding depression is predicted to be elevated in more stressful environments. For questions iv–vi, whether the sexes will differ remains unclear. We determined and statistically accounted for a number of moderators for each effect regarding these factors that could influence sex‐specific inbreeding depression.

## METHODS

### Literature search and inclusion criteria

**FIGURE 1:**
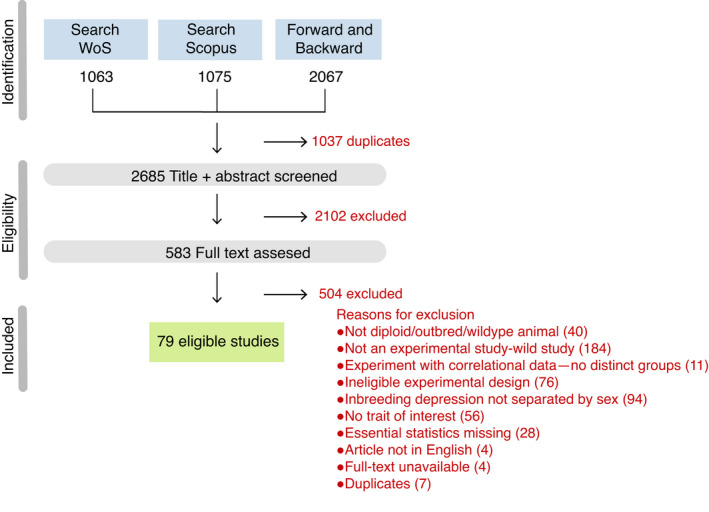
PRISMA diagram describing the search results in Scopus and Web of Science and the different steps of selecting articles for inclusion in the meta‐analysis. Details of each search are provided at https://osf.io/tvx7q/

We searched the published literature for experimental studies in animals that estimated inbreeding depression in males and females. To be able to find relevant terms that could be included in our search, we first made a list of studies that quantified inbreeding depression in males and females based on Ebel and Phillips (2016). We used this study as a reference as it summarised studies that quantified inbreeding depression in both sexes. We also included other studies that could potentially have relevant data based on preliminary searches. We then used the package *scimeetr* in *R* (Rivest, 2018) to find the common keywords used on those studies, and those that would maximise our chances of finding our list of studies in online databases. The procedure used to determine our search terms is available in Supporting Information. We performed a systematic literature search following reporting guidelines from PRISMA‐EcoEvo (Preferred Reporting Items for Systematic Reviews and Meta‐Analyses extension for ecology and evolutionary biology; O'Dea et al., 2021). The PRISMA diagram depicting our search and screening process is shown in Figure 1. We searched the online databases Scopus and Web of Science online in April 2020 accessed through the Stockholm University subscription, containing the following search terms: "male*" OR "female*" OR "sex*" AND "inbreeding depression" OR "coefficient of inbreeding" OR "inbreeding coefficient" OR "inbreeding load" OR "inbreeding affect*" AND NOT "seedling" OR "seed germination" OR "gynodioecy" OR "plant*" OR "pollen" OR "self‐pollination" OR "crop" OR "cattle" OR "livestock" OR "vortex" OR "hymenoptera" OR "patient*" OR "human*" OR "men" OR "women" OR "man" OR "woman" OR "child*" OR "marriage" OR "pig*" OR "horse*" OR "farm" OR "poultry" OR "dog*". Based on the papers that met our criteria for data extraction, we then identified more papers by systematically checking the bibliography from the 10 most recent papers (Backward search) and the 10 most cited papers (Forward search), using again the online databases Scopus and Web of Science. Note that these forward and backward searches were originally planned to include papers that may have addressed sex‐specific inbreeding depression, but which may have been missed due to them specifying only general inbreeding effects, and not sex‐specific effects (particularly when the sex effect was not significant). Duplicates between online databases were first identified using the *revtools* package (v.0.4.1; Westgate, 2019) in *R* (v. 3.6.0; R Development Core Team, 2012), and further duplicates identified in Rayyan software (Ouzzani et al., 2016).

The exact numbers of screened and included studies are shown in Figure 1, and the list of included studies in Supporting Information. We used Rayyan software to screen titles and abstracts (Ouzzani et al., 2016). We screened a total of 2685 unique abstracts. Two people (RV‐T and RdB) screened the abstracts, using a decision tree (Supplementary Section 1). We had a partial overlap of decisions (150 abstracts were initially screened by both people, among which 32 had conflicting decisions, and after resolving these initial conflicting decisions an additional 50 abstracts were screened where 4 were in conflict). Conflicting decisions were discussed and resolved. Approximately 78% of the abstracts were removed because they did not fulfil our inclusion criteria, which left 583 papers for which we read the full text.

We extracted data from studies that met our inclusion criteria. Foremost, an experimental comparison between outbred and inbred individuals was required based on an established pedigree‐based familial relationship. Outbred individuals were assumed to have an *F* (coefficient of inbreeding) of zero, and so studies using inbred strains where “outbred” (i.e. parents unrelated) individuals were used as a reference were excluded as *F* was higher than zero. We focused exclusively on studies that examined inbreeding depression in traits that are present in both sexes and therefore comparable: morphological traits (body size, body mass), mating traits (mating duration, mating latency), reproductive traits (fecundity, paternity success) and survival (or longevity), even if the same study did not test both sexes. We thus targeted studies where inbreeding depression was available for males and females separately and excluded those where the sexes were combined. For example, to compare inbreeding depression in reproductive success between the sexes, we only included effect sizes where one sex at a time was inbred and tested against outbred individuals. That is, inbreeding depression in reproductive success of females would be the effect size of the difference in reproductive success between an inbred female paired with an outbred male compared to an outbred female with an outbred male. We focused on experimental studies and thus excluded non‐experimental field studies and experimental correlational studies (e.g. correlations between an inbreeding coefficient and a trait of interest). Wild studies often use genetic marker‐based pedigrees that provide correlational data that give their own set of limitations as their accuracy depends on the number and variability of markers available (Pemberton, 2008; Taylor, 2015; Taylor et al., 2015), and this was beyond the scope of the current meta‐analysis. Last, a study was only included if it provided sufficient information to estimate an effect size via descriptive or inferential statistics. Where sample sizes or variances were missing, we attempted to contact the authors for this information (*n* = 6). All authors were asked if they could provide additional data that could be used in our analysis. Three out of six authors replied to our data request and these studies were therefore included in our meta‐analysis. A total of 28 studies were excluded because essential statistics were missing. A list of studies included and studies that did not fulfil our selection criteria are listed in the Supporting Information, along with the reason for their exclusion.

### Data extraction and effect size calculation

We quantified inbreeding depression as the difference in fitness components (i.e. measures such as viability, fecundity or mating success; Charlesworth & Charlesworth, 1999) between outbred (*F* = 0) and inbred (*F* = 0.25–0.8) individuals for each male‐male or female‐female combination. Inbreeding depression was coded such that higher values represented higher inbreeding depression (i.e. inbred individuals having lower fitness components). Thus, effect size estimates obtained for relationships such as latency to mate were multiplied by −1 so that positive estimates indicated outbred individuals being of higher ‘quality’.

We collected the means, standard deviations, and sample sizes of outbred—inbred comparisons. Data were extracted from text, tables, figures or raw data. To extract data from figures we used the *metaDigitse* package (v.1.0; Pick et al., 2019) in *R*. We transformed relevant study results into the standardised effect size Hedges *g* (Hedges & Olkin, 1985). Hedges *g* expresses the difference in means in terms of standard deviations, and was used instead of Cohen's *d* because it is more robust to unequal sampling and small sample sizes (Rosenberg et al., 2013). Effect sizes were calculated following the equations in Borenstein et al. (2011). Effect sizes based on test statistics (*t*‐test, chi‐square or *F* values from one‐way Anova's) were converted into Hedges *g* using the equations in Borenstein et al. (2011). See Supporting Information for details on the data processing and the code for extracting means and summary statistics form raw data sets.

### Moderator variables

In addition to the descriptive statistics for inbred and outbred male and females, we extracted the inbreeding coefficient of inbred individuals, the trait measured (categorised in body size, mating, reproduction, survival), and whether individuals were measured under stressful conditions (e.g. competition, food stress, environmental variability, temperature) which was coded as a binary response (1‐stress, 0‐no stress). We additionally included whether the male or the female are the heterogametic sex, and an estimate of sexual size dimorphism (SSD) following (Janicke & Fromonteil, 2021). A description of how the moderators were coded is available in the Supplementary Material. Note that this study was not pre‐registered and as such all moderators were tested using exploratory meta‐regressions.

### Statistical analyses

We fit meta‐analytic and multi‐level meta‐regression models using the *rma*.*mv* function in *R* in the *metafor* package (v. 2.1‐0; Viechtbauer, 2010). All data used in the analyses, plus the R code are available in https://osf.io/tvx7q/. In all models, we incorporated species identity, phylogeny, effect size identity (an observation‐level unique identifier for each effect size calculated), and paper identity as random effects to account for nonindependence of the data from the same studies. Phylogeny was incorporated into all models as a random effect using a variance‐covariance matrix. The phylogeny used to compute a phylogenetic covariance matrix was constructed using the Open Tree of Life package *rotl* (v.3.0.10; Michonneau et al., 2016), and branch lengths were set following Grafen's method (see code in Supporting Information). We estimated heterogeneity in the meta‐analytical model (I) using *I* ^2^ as an estimate of the proportion of variance explained in effect sizes due to differences between levels of a random effect following Nakagawa and Santos (2012). For models that included moderator variables (meta‐regressions—II–XII) we additionally report the marginal *R* ^2^ values (proportion of between‐study variance explained by including the moderator) following Nakagawa and Schielzeth (2010). We obtained point estimates (*ß*) and 95% confidence intervals for each factor level of a moderator from meta‐regressions by removing the intercept. Point estimates were considered statistically significant from 0 when their 95% confidence interval did not overlap zero.

We first calculated the overall mean effect size by running a model with just the random effects listed above. To examine the effect of the moderator variables, we then ran meta‐regressions. Models included the same random effects as above, but now also included each categorical moderator as a fixed effect in separate models (sex, SSD, heterogamety, inbreeding coefficient, trait and stress; models II–VII respectively). Statistical significance of moderators was inferred from ‘omnibus tests’ (‘*Q_m_*’, provided by *metafor*) of meta‐regressions with intercepts. The omnibus test is a Wald‐type *χ* ^2^ test, which tests whether the coefficient(s) of factor level(s) differ from zero (H_0_: *ß*_1_ = *ß_i_* = 0). If the null hypothesis cannot be rejected, it means that none of the factor levels differ from the intercept and the moderator as a whole is not statistically significant (Viechtbauer, 2010). As most of our moderators contain two levels, the omnibus test essentially tested whether one level differed from the other. For moderators with more than two levels, we further examined whether factor levels other than the intercept differed from one another. We did this via linear contrasts in meta‐regressions without an intercept with as null hypothesis H_0_: −*ß_a_* + *ß_b_* = 0. This was achieved with the ‘L’ argument in the ‘anova’ command. To explore the effects of the moderators on each sex, we ran the same models, but this time including an interaction between sex and each one of the moderators (models VIII–XII respectively). To test the statistical significance of the interaction, the omnibus test was used as above but while setting the argument ‘betas to test’ (‘btt’) to the coefficients containing the interaction following the *metafor* package documentation. Combining these fixed factors into a single model could obscure potential effects given the reduction of sample size per category, so emphasis on the significant effects should be interpreted with caution.

### Publication bias and sensitivity analyses

We assessed publication bias in several ways. We first looked for asymmetry in funnel plots by plotting the effect size against precision (i.e. the inverse of standard error; Supplementary Section 14). We then ran Egger's regression on the meta‐analytic residuals (sensu Nakagawa & Santos, 2012) of effect sizes and their sampling errors. These residuals were calculated from the null model that only included random effects using a Bayesian model using the *MCMCglmm* package (v2.29; Hadfield, 2010). Note that we ran the same model using a model with all fixed effects (Supplementary Section 14). This regression tests for deviations in funnel asymmetry, indicated by a slope that differs from 0 (Egger et al., 1997). Next, we used trim‐and‐fill tests to predict ‘missing’ (i.e. unpublished) studies from the literature based on funnel asymmetry (Duval & Tweedie, 2000) by using three estimators *L*_0_, *R*_0_ and *Q*_0_ (Shi & Lin, 2019). We also tested whether studies with larger effects tend to be published earlier (time‐lag effect), by including publication year as a moderator in a meta‐regression model with species, phylogeny, effect size identity and study identity as random effects (Møller & Jennions, 2002). Additional sensitivity analyses were conducted to test whether results changed when estimates were calculated from groups that shared a control group in three different ways. We removed studies with shared controls, used shared control as a moderator, and additionally divided the sample sizes by the number of shared controls and recalculated the overall mean. These models were run for the null model and for the meta‐regression model with sex as a moderator (see Supplementary Section 15). A sensitivity analysis where group identity (effect size estimates from the same where animals were measured more than once) was included as a random effect showed a marginally non‐significant effect of sex (*Q_M_* = 3.77, *df* = 1, *p* = 0.052), but where males and female estimates remained the same as when not including it (Supplementary Section 15). Finally, to test the robustness of our results to the influence of individual studies, we performed leave‐one‐out analyses. To do so, we ran both the meta‐analytical model and the meta‐regression model that included sex as a moderator iteratively. That is, removing one study at a time to ensure our findings were not driven by a single study (Baldwin et al., 2012; Viechtbauer, 2010). All data and code are made available at https://osf.io/tvx7q/.

## RESULTS

### The effect size dataset

**FIGURE 2:**
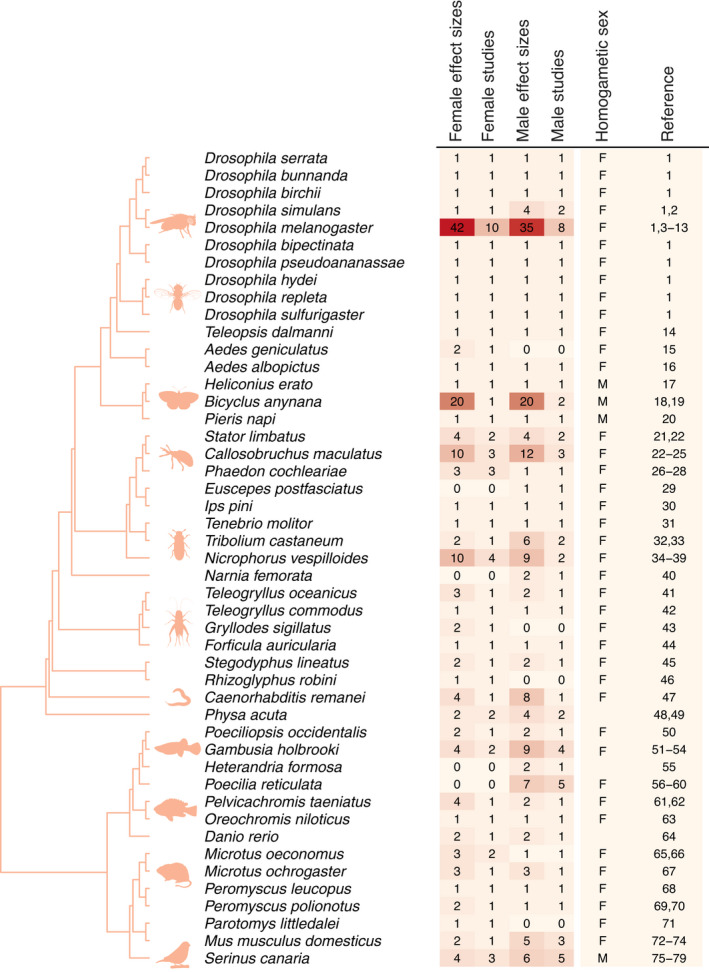
Phylogenetic distribution of the species included in the meta‐analysis. Primary study references and graphical summary of the phylogenetic distribution. Depicted are phylogenetic relatedness, scientific names, number of effect sizes for males and females, number of studies for each species and sex, and the homogametic sex for each species (F = female, M = male). Larger numbers of effect sizes are highlighted with darker colouration. Animal silhouettes obtained from phylopic.org. References: 1: Bechsgaard et al. (2013); 2: Okada et al. (2011); 3: Radwan and Drewniak (2001); 4: Charlesworth et al. (2007); 5: Enders and Nunney (2010); 6: Ala‐Honkola et al. (2013); 7: Long et al. (2013); 8: Mazzi et al. (2013); 9: Ala‐Honkola et al. (2013); 10: Ala‐Honkola et al. (2014); 11: Ala‐Honkola et al. (2015); 12: Dolphin and Carter (2016); 13: Enders and Nunney (2016); 14: Prokop et al. (2010); 15: Armbruster et al. (2000); 16: O'Donnell and Armbruster (2010); 17: De Nardin et al. (2016); 18: Joron and Brakefield (2003); 19: Dierks et al. (2012); 20: Välimäki et al. (2011); 21: Fox and Scheibly (2006); 22: Fox et al. (2006); 23: Fox and Stillwell (2009); 24: Fox et al. (2012); 25: Messina et al. (2013); 26: Müller and Jukauskas (2018); 27: Müller et al. (2018); 28: Muller et al. (2018); 29: Kuriwada et al. (2011); 30: Domingue and Teale (2007); 31: Rantala et al. (2011); 32: Pray et al. (1994); 33: Michalczyk et al. (2010); 34: Pilakouta and Smiseth (2017); 35: Richardson and Smiseth (2017); 36: Ford et al. (2018); 37: Mattey et al. (2018); 38: Ratz et al. (2018); 39: Richardson et al. (2018); 40: Joseph et al. (2016); 41: Simmons (2011); 42: Drayton et al. (2011); 43: Sakaluk et al. (2019); 44: Meunier and Kolliker (2013); 45: Bilde et al. (2005); 46: Radwan (2003); 47: Ebel and Phillips (2016); 48: Janicke et al. (2013); 49: Janicke et al. (2014); 50: Sheffer et al. (1999); 51: Vega‐Trejo, Head, et al. (2016); 52: Vega‐Trejo, Jennions, et al. (2016); 53: Marsh et al. (2017); 54: Vega‐Trejo et al. (2017); 55: Ala‐Honkola et al. (2009); 56: Sheridan and Pomiankowski (1997); 57: Mariette et al. (2006); 58: Pitcher et al. (2008); 59: Zajitschek and Brooks (2010); 60: Gasparini et al. (2013); 61: Langen et al. (2017a); 62: Langen et al. (2017b); 63: Fessehaye et al. (2009); 64: Bickley et al. (2013); 65: dos Santos et al. (1995); 66: Gundersen et al. (2001); 67: Lucia‐Simmons and Keane (2015); 68: Jimenez et al. (1994); 69: Margulis (1998); 70: Margulis and Walsh (2002); 71: Pillay and Rymer (2017); 72: Eklund (1996); 73: Meagher et al. (2000); 74: Ilmonen et al. (2009); 75: de Boer et al. (2015); 76: de Boer et al. (2016b); 77: de Boer et al. (2016a); 78: de Boer et al. (2018b); 79: de Boer et al. (2018a)

The final data set included 321 effect sizes across 79 studies and 47 species. Figure 2 shows the spread of data across the phylogeny. We obtained roughly the same number of effect sizes between the sexes: 153 effect sizes for females (48%), and 168 effect sizes for males (52%). Of those studies, 37 included effect sizes for both sexes in the same study, with 96 effect sizes for females and 98 for males. Most effect size estimates (72%) were obtained from studies with an inbreeding coefficient *F* = 0.25 (i.e. offspring resulting from matings between individuals related at the full‐sibling level, *r* = 0.5). We retrieved 138 effect sizes associated with reproduction, 82 with body size, 71 for survival, and 30 with mating (see Supplementary Section 14 for details on how they were distributed between the sexes). Our dataset included species from four animal phyla (Arthropoda, Chordata, Mollusca, Nematoda), but were mostly represented by insects (70%, Supplementary Section 10). Biological inherent differences associated with animal class, reproductive mode (internal vs. external fertilizers), or reproductive mode (egg‐laying vs. live‐bearing species) were explored but were found to have no effect (Supplementary Sections 10–12).

### Sex‐specific inbreeding depression

**FIGURE 3:**
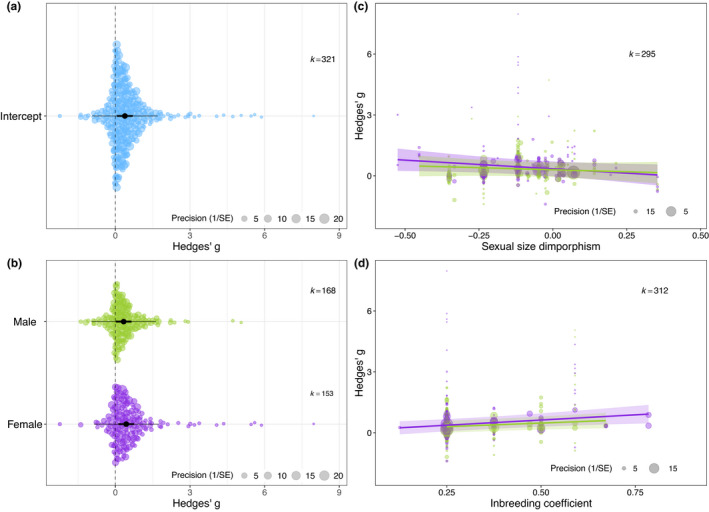
Meta‐regression means of (a) meta‐analytical mean of inbreeding depression, (b) meta‐regression of the effect of sex, (c) meta‐regression of the effect of sexual size dimorphism, and (d) meta‐regression of the effect of the inbreeding coefficient. The size of each data point represents the precision of the study (1/SE). (a) and (b) depict orchard plots where the position of the data point on the *x*‐axis represents the effect size value, and the position on the *y*‐axis is spread out randomly depending on the density of points at any given *x*‐value. The meta‐analytic means with 95% confidence intervals are depicted in black and the 95% prediction intervals in grey. In (c) and (d) the 95% confidence intervals of the regression lines between the inbreeding coefficient and effect size estimates are shown in a lighter colour. Males are shown in green and females in purple

Overall, we found evidence for inbreeding depression as the grand mean across all types of effect sizes was positive (*β* [95% CI] = 0.38 [0.06; 0.7], *Z* = 2.34, *k* = 317, *p* = 0.02). The total heterogeneity (*I* ^2^) among the random factors was 93.19%; with most of the variance attributed to between‐species differences (47.3%), while phylogeny, variance attributed to between‐study differences, and observational study‐differences were similar (13.61%, 15.9% and 16.38% respectively). We found evidence of sex‐specific inbreeding depression, with a (weak) difference in overall inbreeding depression between males and females (*Q_M_* = 4.16, *df* = 1, *p* = 0.041; Table 2). Both males and females showed inbreeding depression, but females showed a stronger effect than males (females estimate [95% CI] = 0.44 [0.12; 0.75]; males estimate [95% CI] = 0.33 [0.02; 0.65]; Table 2, Figure 3b). Including sex as a moderator explained very little heterogeneity (*R* ^2^ = 0.6%). Note that this effect was similar when only including species where estimates were available for both sexes and under the same conditions (36 species, *Q_M_* = 4, *df* = 1, *p* = 0.045, females estimate [95% CI] = 0.41 [0.19; 0.63]; males estimate [95% CI] = 0.29 [0.07; 0.51]; Supplementary Section 15).

**TABLE 2:** Meta‐analyses assessing sex‐specific effects of inbreeding depression. (I) Overall meta‐analytical estimate. (II–XII) moderators of sex‐specific inbreeding. For each meta‐regression the levels of the moderators are indicated, along with number of effect sizes (*k*) for each level in the moderator, the number of species included for each level in the moderator (spp.), the total number of effect sizes (*k*_total_), the marginal explained variation by the moderator (*R* ^2^_marg_), and the model degrees of freedom (*df*). Omnibus *Q* test of moderators (*Q_m_*), and the *p*‐value (*p*) are noted from models with the intercept. Significant *p*‐values and estimates that differ from zero are indicated in bold text. Point estimates (*β*) and 95% confidence intervals from models without intercepts are also shown. Note that the inbreeding coefficient and sexual size dimorphism were continuous moderators and that point estimates for these models correspond to models with the intercept. (*Q_m_*) and *p*‐values for interaction terms are given by testing the coefficients containing the interaction (details in the Methods)

Moderator of inbreeding depression	Moderator levels (*k*) and number of species (spp.)	*k*_total_	*R* ^2^_mar_(%)	*df*	*Q_m_*	*p*	*β*	95% CI
(I) Meta‐analytical model	*k* = 321, spp. = 47	321	—	—	—	**0.02**		
(II) Sex	Female (*k* = 153, spp. = 43) Male (*k* = 168, spp. = 43)	321	0.64	1	4.16	**0.041**	0.44 0.33	**[0.12; 0.75]** **[0.02; 0.65]**
(III) Sexual size dimorphism		295	4.94	1	2.22	0.136	−0.66	[−1.53; 0.21]
(VIII) Sex × Sexual size dimorphism		295	3.43	1	1.41	0.234		
(IV) Heterogamety	Heterogametic (*k* = 151, spp. = 38) Homogametic (*k* = 150, spp. = 38)	309	0.08	1	0.55	0.459	0.39 0.43	**[0.06; 0.72]** **[0.1; 0.76]**
(IX) Sex × Heterogamety		301	0.57	1	0.01	0.942		
(V) Inbreeding coefficient		312	2.38	1	8.49	**0.004**	0.84	**[0.28; 1.41]**
(X) Sex × Inbreeding coefficient		312	3	1	0.65	0.422		
(VI) Trait	Body size (*k* = 82, spp. = 27) Mating (*k* = 30, spp. = 10) Reproduction (*k* = 138, spp. = 29) Survival (*k* = 71, spp. = 11)	321	1.2	3	3.35	0.341	0.33 0.46 0.45 0.29	**[0.01; 0.66]** **[0.1; 0.81]** **[0.12; 0.78]** [−0.07; 0.64]
(XI) Sex × Trait		321	2.38	3	5.14	0.162		
(VII) Environmental stress	No stress (*k* = 219, spp. = 45) Stress (*k* = 102, spp. = 14)	321	3	1	0.37	0.544	0.39 0.35	**[0.07; 0.71]** **[0.02; 0.68]**
(XII) Sex × Environmental stress		321	0.71	1	0.01	0.93		

### Additional factors hypothesised to affect inbreeding depression

#### Sexual size dimorphism

We did not find a relationship between SSD and effect size estimates of inbreeding depression (*β* [95% CI] = −0.66 [−1.53; 0.21]; Table 2). Note that when including insects only (as SSD tends to be female size biased), there was no relationship between SSD and effect size estimates either (*β* [95% CI] = −1.44 [−3.23; 0.36]; Supplementary Section 5). Nor did we find an interaction between sex and SSD (Table 2, Figure 3c; Supplementary Section 14).

#### Homo‐/heterogamety

**FIGURE 4:**
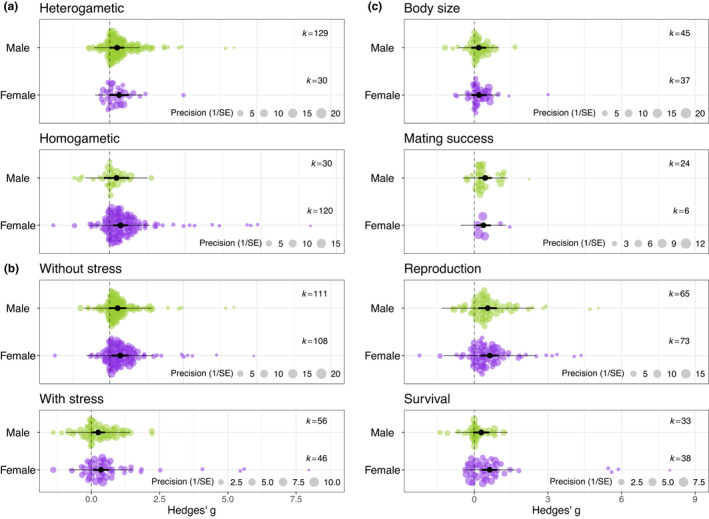
Meta‐regression means of (a) heterogamety, (b) environmental stress, and (c) type of traits on sex‐specific inbreeding depression. Orchard plots from models where each level was tested separately with sex as a moderator and where the position of the data point on the *x*‐axis represents the effect size value, and the position on the *y*‐axis is spread out randomly depending on the density of points at any given *x*‐value. The size of each data point represents the precision of the study (1/SE). The meta‐analytic means with 95% confidence intervals are depicted in black and the 95% prediction intervals in grey. Males are shown in green and females in purple

Homogametic species did not differ from heterogametic species in inbreeding depression (Table 2), nor was there an interaction between heterogamety and sex (Table 2; Figure 4a). Note that we collected a higher number of effect sizes for homogametic females due to the majority of our data including XY systems, where females are the homogametic sex (74%; Supplementary Sections 6 and 14), and that 4% of the effect sizes were not assigned heterogamety due to a lack of information or ambiguity on determining heterogamety for some species (*Physa acuta*, *Heterandria formosa*, *Danio rerio*).

#### Coefficient of inbreeding

We found a positive correlation between the coefficient of inbreeding (when treated as a continuous covariate) and effect size estimates of inbreeding depression (*β* [95% CI] = 0.85 [0.27; 1.41]; Table 2), although it explained a small proportion of variation relative to the total amount of variation (*R* ^2^ = 2%). When exploring this effect further, we did not find a significant interaction between sex and the inbreeding coefficient (Table 2; Figure 3d).

#### Traits assessed

There was no difference between effect sizes when inbreeding depression was measured for body size, mating success, reproduction or survival (*Q_m_* = 3.35, *k* = 321, *df* = 3, *p* = 0.341; Table 2; Supplementary Section 8). There was also no significant interaction between the type of trait measured and sex (Table 2, Figure 4c; Supplementary Section 14), although when only considering survival effect size estimates with sex as a moderator, effect sizes differed between males and females (*Q_m_* = 12.78, *k* = 69, *df* = 1, *p* < 0.001; Supplementary Section 9).

#### Environmental stress

Effect size estimates when measured with or without stress did not differ from each other (*Q_m_* = 0.37, *k* = 321, *df* = 1, *p* = 0.544; Table 2). There was also no significant interaction between the type of trait measured and sex (Table 2, Figure 4b; Supplementary Section 14). There was an imbalance in the number of estimates we were able to obtain that measured traits with and without stress. Most effect sizes (68%) came from inbreeding depression quantified under standard conditions without environmental stress (Supplementary Section 9). Note, however, that when considering effect size estimates measured under stress, they did not differ from each other due to the type of stress (*Q_m_* = 2.46, *k* = 102, *df* = 3, *p* = 0.483; Supplementary Section 9).

### Publication bias

Visual inspection of funnel plots indicated some asymmetrical distribution of effect sizes around the meta‐analytical mean (Supplementary Section 16), with small, imprecise studies that show inbreeding depression being more abundant. The results of Egger's regression on the meta‐analytic residuals confirmed the presence of publication bias in the data set (Intercept ± SE: 0.73 ± 0.21, *t* = 3.5, *p* < 0.001; Supplementary Section 16). To test the sensitivity of our meta‐analytical mean, we used trim‐and‐fill tests. When accounting for these tests, the meta‐analytical mean appeared fairly robust (Supplementary Section 16). Some effect sizes had shared control groups (i.e. the same group of animals served as control group for both the inbred and outbred individuals). We ran a sensitivity analysis for the inclusion of shared controls because the true sample size is lower for these effect sizes than for those with no shared control groups. Having a shared control group did not have an effect on the meta‐analytical mean (Supplementary Section 15). However, although including shared control as a moderator in the meta‐regression with sex in the model had no effect, it did affect the difference between the sexes. This difference was no longer significant, although females being different from zero remained the same (females estimate [95% CI] = 0.43 [0.09; 0.78]; males estimate [95% CI] = 0.38 [0.04; 0.73]; Supplementary Section 15). Last, we found no significant relationship between study year and effect sizes (Supplementary Section 16).

### Leave‐one‐out sensitivity analyses

One study in our dataset (Bechsgaard et al., 2013) appeared to have a large effect and so to test the sensitivity of our meta‐analytic mean and meta‐regression means to this study, we re‐ran the whole analyses without this study. The main difference was sex differences in inbreeding depression were more noticeable, as the meta‐regression of sex was significant, opposed to marginally significant (*Q_m_* = 5.31, *df* = 1, *p* = 0.021, *R* ^2^ = 1.63%; females estimate [95% CI] = 0.39 [0.15; 0.63]; males estimate [95% CI] = 0.27 [0.04; 0.51]; Supplementary Section 14). When excluding this study, there was a weak effect for differences between animal classes (*Q_m_* = 9.74, *df* = 4, *p* = 0.045, *R* ^2^ = 16.68%), with insects showing inbreeding depression (estimate [95% CI] = 0.49 [0.28; 0.69]), a trend that was evident on the full dataset. Note that this study focused on different *Drosophila* species, highlighting the taxonomic bias found in the current meta‐analysis. The results of these analyses are provided at https://osf.io/tvx7q/.

## DISCUSSION

We found evidence for general inbreeding depression for both males and females and, although the difference between the sexes was not substantial, females appeared to be slightly more sensitive to inbreeding depression than males. We also found that the severity of inbreeding depression was associated with the degree of inbreeding (i.e. the inbreeding coefficient). As there is a taxonomic bias in our study—arthropods were overrepresented in our data—evidence from additional species is necessary in order to generalize this result. Our study highlights the need to further explore the effects of inbreeding depression due to intrinsic biological differences between males and females.

Could sexual selection explain the differences between male and female sensitivity to inbreeding depression? Sexual selection is expected to purge deleterious mutations more efficiently in males as males generally experience more intense sexual selection than females (Andersson, 1994). Because sexual selection theory predicts females to choose ‘superior’ males, being inbred may be more costly for males than for females (Enders & Nunney, 2010). As a consequence, deleterious mutations could be purged in males through sexual selection (Ebel & Phillips, 2016). Moreover, a recent meta‐analysis found that sexual selection tends to increase population mean values for female fitness traits (Cally et al., 2019). Our results indicate that although males do show inbreeding depression, the effect in females tends to be stronger. Note, however, that the variance explained by sex in the dataset was very low (0.62%), and thus the generality of this effect should be taken with caution. In addition, it is hard to make general predictions about the strength of inbreeding depression between the sexes when not accounting for sexual selection given this may lead to greater inbreeding depression in males (Enders & Nunney, 2010; Janicke et al., 2013), and because the effect of sexual selection can vary between environments (Cally et al., 2019). When testing if SSD, as a proxy for sexual selection, was related to effect size estimates of inbreeding depression, we found no such relationship potentially because SSD may not be a good predictor for pre‐copulatory sexual selection across taxa (Janicke & Fromonteil, 2021). Indeed, as a generalised proxy for ‘strength of sexual selection’ among studies is challenging to obtain, few studies included such a proxy explicitly. A key avenue for future research would involve varying levels of sexual selection to determine each of the sexes sensitivity to inbreeding depression (Carazo et al., 2016), particularly under scenarios that reflect species' natural conditions.

Contrary to expectations, the higher sensitivity to inbreeding depression we observed in females does not appear to be explained by heterogamety. Species differ in their chromosomal content, with most insects and mammals having homogametic females (XX), while birds and butterflies having heterogametic females (ZW). Differences in the expression of genes and selection pressures experienced by individuals may build up the inbreeding load differently by each sex (i.e. differences between the sexes of the rate at which fitness declines with increased inbreeding coefficient; Charlesworth & Willis, 2009; Vermeulen et al., 2008). For instance, when males are the heterogamous sex (i.e. species with XY or ZW determination), genes on the X chromosome are always effectively dominant, but may be recessive in females and contribute to inbreeding depression in females (Ebel & Phillips, 2016). Differences in lifespan between the sexes have been associated with a reduction or absence chromosome in the heterogametic sex (the unguarded X hypothesis; Trivers, 1985), where the homogametic sex lives longer than the heterogametic sex (Xirocostas et al., 2020). However, we found no difference between hetero‐ and homogametic species, and no interaction with sex. Thus, our results provide no support for the hypothesis that heterogamety underlies sex‐specific inbreeding depression. This is in line with previous studies which also concluded that heterogamy does not adequately explain sex‐specific differences in inbreeding depression (Bilde et al., 2009; Saccheri et al., 2005).

Inbreeding depression was stronger at higher levels of inbreeding as we found a positive relationship between inbreeding level and inbreeding depression. This was expected, as the costs of inbreeding depression are predicted to be higher when the coefficients of inbreeding are highest (Charlesworth & Willis, 2009). Purging for deleterious alleles with an increasing level of inbreeding is likely. However, we still observed inbreeding depression in both sexes at high inbreeding coefficients. Mutational load has greater potential to be purged via selection in males, owing to sexual selection operating more strongly in males, particularly where condition‐dependent selection is strong (Grieshop et al., 2021; Rowe & Houle, 1997; Whitlock & Agrawal, 2009). However, the relative strength of purging selection in males versus females might vary depending on environmental conditions (Plesnar‐Bielak et al., 2020). Moreover, experimental evidence has shown only limited effects of purging on fitness restoration and its efficiency may depend on the nature of genetic variance (Frankham, 2015; Wright et al., 2007). It remains to be explored how experimental conditions may change purging in both males and females.

There was no difference in the magnitude of inbreeding depression between the traits we compared. However, there was a trend for females to suffer stronger declines in survival than males. This could lead to a stronger overall cost of inbreeding for females than for males. Inbreeding depression being worse in females than in males for lifespan has indeed been shown in *Drosophila* (Sultanova et al., 2018). Body size did not show inbreeding depression for either sex, although larger body sizes are often associated with larger reproductive success (Kingsolver & Pfennig, 2004). As such, inbreeding may impair mating success in a body‐size independent manner and further work that explores their link and the underlying traits that lead to a reduced reproductive success is needed. Moreover, this may be due to fitness‐related traits such as survival and reproduction having a relatively large component of directional dominance, as opposed to traits under more weak or balancing selection (Lynch & Walsh, 1998; Saccheri et al., 2005). The lack of differences in reproduction could also be due to how genes and adaptations for fecundity differ between the sexes and because intra and intersexual selection may play a larger role in males (Carazo et al., 2016; Sultanova et al., 2018) which we were not able to detect. Although sex differences in inbreeding depression could be associated with sex‐specific effects of loci affecting allocation of resources between reproduction and somatic maintenance (Fox et al., 2006), whether this reallocation of resources could indeed generate differences between the sexes needs to be further explored.

Deriving estimates of selection on both sexes are challenging given the complexity of comparing genotypes in each sex and traits being measured. Of particular importance is the environment that individuals experience. Although inbreeding depression is expected to increase under stressful environments (Armbruster & Reed, 2005; Fox & Reed, 2011), and the strength of inbreeding depression can differ between the sexes under stressful conditions (e.g. Janicke et al., 2013), inbreeding depression was not higher under stressful environments in our analysis. We recognise that controlled environments in which experimental studies are conducted, may not always capture ecologically relevant conditions in nature. Additionally, laboratory‐adapted populations frequently experience bottleneck events during their establishment or maintenance (Enders & Nunney, 2010). As such, experimental studies offer a great value to understand the effects of inbreeding depression, but also have their limitations. We suspect however, that the lack of a stronger effect of inbreeding depression under stressful environments is due to the low number of estimates under stressful conditions, compared to those under no stress. Moreover, different kinds of stress were grouped into one category only due to the low number of different types of stress, which could obscure stress effects. Having more estimates under stress would also allow to refine the type of environmental stress and would allow for an analysis in which stress could be categorised from low to high.

The exact mechanism(s) underlying sex‐specific inbreeding depression, and why females may be more sensitive remains elusive. Nevertheless, there could be far‐reaching consequences of sex‐specific inbreeding depression. Population growth is often constrained by the number of females, not males, and if indeed females suffer more from inbreeding depression, this may in fact be beneficial for population growth if deleterious alleles could be exposed and be purged faster in females. However, purging being higher in females was not supported in our data as we found inbreeding depression to be slightly higher in females. Conversely, female inbreeding depression may limit population growth as the detrimental effects of inbreeding may affect how much they can invest in reproduction. This may be particularly relevant in small populations where inbreeding is more likely; a key link to be explored in future research. Another consequence is that females may be more driven to avoid inbreeding than males, potentially modulating sexual conflict. This could be linked to females typically making a higher investment per offspring when compared to males (Kokko & Jennions, 2003), and the corresponding increase in female mutational load (Bonduriansky & Chenoweth, 2009; Connallon & Clark, 2011; Day & Bonduriansky, 2004). Although a recent meta‐analysis did not find sex‐specific effects of inbreeding avoidance (de Boer et al., 2021), it was pointed out that this should be examined within a species, similar to sex‐specific inbreeding depression. As such, a fruitful avenue for further research is to quantify inbreeding depression in males and females and to see how this modulates sexual conflict over mating and inbreeding. The generality of females being more sensitive to inbreeding depression should be taken with caution as we found evidence of publication bias, whereby studies showing inbreeding depression (i.e. positive findings) for both sexes were more likely. This may be associated with the directionality of effect sizes and the likelihood of papers being accepted for publication when they follow a particular ‘expected’ direction (Jennions et al., 2013). Furthermore, published effect sizes tend to be larger than unpublished ones (Kim et al., 2021; Sánchez‐Tójar et al., 2018), and the fact that we did not include unpublished effect sizes may have influenced our results.

The mechanisms behind sex‐specific inbreeding depression may be too system‐specific to identify general patterns. For example, the effects of inbreeding depression may vary with population demography, genetic composition, stress and levels of sexual conflict across species or even populations. Such system‐specific variance makes generating predictions challenging. However, by comparing different species we have tried to identify some of the main causes of differences in inbreeding depression between males and females. The taxonomic gaps in the literature are reflected in our meta‐analysis, with a general focus on model systems (e.g. *Drosophila*) which are easy to culture, may be well‐adapted lab populations, and where measuring traits in both sexes is easier (and faster) than in other systems. Consequently, there was also bias for internal over external fertilisers and egg‐laying over live‐bearing species. The difficulty of assessing fitness in adult traits in both sexes further complicates our ability to understand how inbreeding impairs performance in males and females. Given the somewhat limited taxonomic breadth of effect sizes included in our study, our results should be interpreted with caution. For general patterns across animals to be apparent, more studies are needed, particularly so in vertebrates. This will help us gain a broader perspective about the nature of inbreeding depression by examining a larger set of species and under variable environments.

There seem to be many confounding factors that are yet to be determined to be able to ascertain the effects of inbreeding depression in males and females. For example, most species included in our study are female homogametic systems (i.e. XX). More empirical data from heterogametic systems is therefore needed before heterogametic effects in sex‐specific inbreeding depression can be ruled out conclusively. Moreover, studies investigating inbreeding depression in a specific environment cannot be used to infer the long‐term effects of inbreeding (Kristensen et al., 2008), so there is a need for studies that examine effects across lines, sexes and traits in variable environments. Understanding sex‐specific patterns will be key to predict how males and females respond to inbreeding and consequently the evolutionary dynamics of populations (Ebel & Phillips, 2016). In conclusion, our findings indicate that females are more sensitive to inbreeding depression but highlight key research gaps that need to be addressed before being able to generalise these findings.

## AUTHOR CONTRIBUTIONS

RV‐T and RAdB designed the study, collected the data, performed the analyses and wrote the first draft. AK and JLF edited further versions of the manuscript.

## COMPETING INTERESTS

The authors declare no competing interests.

### PEER REVIEW

The peer review history for this article is available at https://publons.com/publon/10.1111/ele.13961.

## Supporting information

Supplementary Material

## Data Availability

All data and code are available at https://osf.io/tvx7q/.
