# Supplementary material for: Sex‐specific inbreeding depression: A meta‐analysis
Source: Ecol Lett. 2022 Jan 21;25(4):1009–26. doi: 10.1111/ele.13961 (PMC9304238; doi:10.1111/ele.13961)
Supplement: Supplementary file 1 — Supplementary Material [file ELE-25-1009-s001.pdf]

Sex-specific inbreeding depression: A meta-analysis

Regina Vega-Trejo, Raïssa A. de Boer, John L. Fitzpatrick, Alexander Kotrschal

Section I. Moderators included in the meta-analysis.

Ia. Descriptions of continuous moderators for meta-regression models.

Continuous moderator	Description
Sexual size dimorphism	Males are generally expected to be under stronger sexual selection due to sex-specific differences in gametic investment (i.e., anisogamy, Parker *et al.* 1972) and because males typically experience greater variance in reproductive success than females (Wade 1979; Janicke *et al.* 2016). Deleterious alleles exposed to inbreeding may make males more sensitive to inbreeding depression either through purging or directional dominance being stronger on males (Wolak & Keller 2014; Grieshop *et al.* 2021).<br><br>Body size is considered a sexually selected trait as it may confer an advantage during episodes of sexual selection (pre, postcopulatory) and can be used as a proxy for sexual selection (Janicke & Fromonteil 2021).<br><br>Sexual size dimorphism for the species included in the study was calculated following Janicke and Fromonteil (2021). To do so, body size (body length, body mass, thorax length, wing length, elytra length, pronotum length) was used to calculate sexual size dimorphism:<br><br>$$SSD = ln\left(\frac{male\ size}{female\ size}\right)$$<br><br>where positive or negative values indicate species with males or females being the larger sex, respectively.
Inbreeding coefficient	Coefficient of inbreeding (probability of two alleles at a random chosen locus being identical by descent).<br>Comparison of outbred F = 0 vs inbred individuals F = 0.125 - 0.785.<br>The costs of inbreeding depression are predicted to be higher when the coefficients of inbreeding are highest (Charlesworth & Willis 2009), and so individuals with a higher inbreeding coefficient were expected to show stronger inbreeding depression.
Publication Year	Year of publication, as extracted from Web of Science or Scopus.

Ib. Description of categorical moderators for meta-regression models.

Categorical moderator	Category	Description
Sex		Males and females can be differently affected by inbreeding depression. Different processes may lead to non-mutually exclusive outcomes (sexual selection, heterogamety, dominance, sexual conflict) as to which sex may be more sensitive to inbreeding depression.
	Male	Trait measured in male individuals.
	Female	Trait measured in female individuals.
Heterogamety		Sex chromosomes are heteromorphic in many species, leading to one sex carrying a heterogamous set of sex chromosomes (e.g. XY males in XY systems and ZW females in ZW systems) and the other a homozygous set (e.g. XX females in XY systems and ZZ males in ZW systems). Genes on the heterozygous sex chromosomes (e.g. XY males) cannot contribute to inbreeding depression, because deleterious recessive alleles on the sex chromosome cannot be masked by a second dominant allele and are thus expressed regardless of the degree of inbreeding (Agrawal 2011; Sultanova *et al.* 2018).
	Homogametic	Sex where sex chromosomes are the same (e.g. XX, ZZ).
	Heterogametic	Sex where sex chromosomes are not the same (e.g. XY, ZW).
Stress		Inbreeding depression is expected to be exacerbated under stressful conditions (Armbruster & Reed 2005; Fox & Reed 2011). Stronger sexual selection on males is often associated with density dependence, which reflects more intense intraspecific competition (i.e., a stressful environment, Yun & Agrawal 2014). Yet, selection in males and females can align under stressful conditions (Martinossi-Allibert *et al.* 2017).<br><br>Note that stressful conditions were determined as stressful based the description provided by the authors of the papers included in our data, and in most cases, there was a comparison between outbred and inbred individuals that were in stressful vs non stressful environments.
	No	Traits were measured under non-stressful environments
	Yes	Traits were measured under stressful environments (temperature stress, chemical stress, competition, food stress, environmental complexity).
Traits		The effects of inbreeding depression on different types of traits will differ due to the genetic architecture of the traits (Bolund *et al.* 2010). For instance, life history traits and sexually selected traits are expected to suffer higher levels of inbreeding depression than morphological traits (Crnokrak & Roff 1999; Cotton *et al.* 2004). The interplay between directional selection and sex-specific inbreeding depression is not known, and therefore fitness related traits were examined.
	Body size	Effect size measured in body size or body weight.
	Mating	Effect size measured in number of copulations, mating duration, mating latency.
	Reproduction	Effect size measured in number of offspring, paternity success, fecundity, egg viability.
	Survival	Effect size measured in lifespan or longevity.

Class		Taxonomic class species belong to. Much information on inbreeding depression come from model species, and is essential to know how general are patterns across taxonomic groups.
	Actinopterygii	
	Arachnida	
	Aves	
	Gastropoda	
	Insecta	
	Mammalia	
	Secernentea	
	Teleostei	
Fertilisation mode		Differences in the site of fertilisation may predict the extent of inbreeding depression and in turn affect the evolution of mating systems (Cohen 1996). External fertilisers share key life history traits with seed plants (low mobility, release of gametes into the environment) (Olsen *et al.* 2020), which may lead to external fertilisers being more sensitive to inbreeding depression.
	Internal	Trait measured in species with internal fertilisation.
	External	Trait measured in species with external fertilisation.
Reproductive mode		The mode of reproduction can affect how sensitive species are to inbreeding depression as allocation and investment of resources may differ. Whether males or females could be differentially affected by this inherent biological difference has not been addressed.
	Egg-laying	Trait measured in species that lay eggs.
	Live-bearing	Trait measured in species that give birth to live young.

References

Agrawal, A.F. (2011). Are males the more 'sensitive' sex? *Heredity (Edinb)*, 107, 20-21.

Armbruster, P. & Reed, D.H. (2005). Inbreeding depression in benign and stressful environments. *Heredity (Edinb)*, 95, 235-242.

Bolund, E., Martin, K., Kempenaers, B. & Forstmeier, W. (2010). Inbreeding depression of sexually selected traits and attractiveness in the zebra finch. *Animal Behaviour*, 79, 947-955.

Charlesworth, D. & Willis, J.H. (2009). The genetics of inbreeding depression. *Nature Reviews: Genetics*, 10, 783-796.

Cohen, C.S. (1996). THE EFFECTS OF CONTRASTING MODES OF FERTILIZATION ON LEVELS OF INBREEDING IN THE MARINE INVERTEBRATE GENUS CORELLA. *Evolution*, 50, 1896-1907.

Cotton, S., Fowler, K. & Pomiankowski, A. (2004). Do sexual ornaments demonstrate heightened condition-dependent expression as predicted by the handicap hypothesis? *Proceedings of the Royal Society B-Biological Sciences*, 271, 771-783.

Crnokrak, P. & Roff, D.A. (1999). Inbreeding depression in the wild. *Heredity*, 83, 260-270.

Fox, C.W. & Reed, D.H. (2011). Inbreeding depression increases with environmental stress: an experimental study and meta-analysis. *Evolution*, 65, 246-258.

Grieshop, K., Maurizio, P.L., Arnqvist, G. & Berger, D. (2021). Selection in males purges the mutation load on female fitness. *Evolution Letters*, 5, 328-343.

Janicke, T. & Fromonteil, S. (2021). Sexual selection and sexual size dimorphism in animals. *Biology Letters*, 17, 20210251.

Janicke, T., Häderer, I.K., Lajeunesse, M.J. & Anthes, N. (2016). Darwinian sex roles confirmed across the animal kingdom. *Science Advances*, 2, e1500983.

Martinossi-Allibert, I., Arnqvist, G. & Berger, D. (2017). Sex-specific selection under environmental stress in seed beetles. *Journal of Evolutionary Biology*, 30, 161-173.

Olsen, K.C., Ryan, W.H., Winn, A.A., Kosman, E.T., Moscoso, J.A., Krueger-Hadfield, S.A. *et al.* (2020). Inbreeding shapes the evolution of marine invertebrates. *Evolution*, 74, 871-882.

Parker, G.A., Baker, R.R. & Smith, V.G.F. (1972). The origin and evolution of gamete dimorphism and the male-female phenomenon. *Journal of Theoretical Biology*, 36, 529-553.

Sultanova, Z., Andic, M. & Carazo, P. (2018). The "unguarded-X" and the genetic architecture of lifespan: Inbreeding results in a potentially maladaptive sex-specific reduction of female lifespan in Drosophila melanogaster. *Evolution*, 72, 540-552.

Wade, M.J. (1979). Sexual Selection and Variance in Reproductive Success. *The American Naturalist*, 114, 742-747.

Wolak, M.E. & Keller, L.F. (2014). Dominance genetic variance and inbreeding in natural populations. In: *Quantitative Genetics in the Wild* (eds. Charmantier, A, Garant, D & Kruuk, LEB). Oxford University Press, Oxford, pp. 104-128.

Yun, L. & Agrawal, A.F. (2014). Variation in the strength of inbreeding depression across environments: Effects of stress and density dependence. *Evolution*, 68, 3599-3606.

Section II. List of species included in the meta-analysis with their corresponding heterogamy.

Species	Female	Male	Family	Order	Class	Heterogametic system	Source
Drosophila serrata	homogametic	heterogametic	Drosophilidae	Diptera	Insecta	XX, XY	(Navara 2018)
Drosophila bunnanda	homogametic	heterogametic	Drosophilidae	Diptera	Insecta	XX, XY	(Navara 2018)
Drosophila birchii	homogametic	heterogametic	Drosophilidae	Diptera	Insecta	XX, XY	(Navara 2018)
Drosophila simulans	homogametic	heterogametic	Drosophilidae	Diptera	Insecta	XX, XY	(Navara 2018)
Drosophila melanogaster	homogametic	heterogametic	Drosophilidae	Diptera	Insecta	XX, XY	(Navara 2018)
Drosophila bipectinata	homogametic	heterogametic	Drosophilidae	Diptera	Insecta	XX, XY	(Navara 2018)
Drosophila pseudoananassae	homogametic	heterogametic	Drosophilidae	Diptera	Insecta	XX, XY	(Navara 2018)
Drosophila repleta	homogametic	heterogametic	Drosophilidae	Diptera	Insecta	XX, XY	(Navara 2018)
Drosophila sulfurigaster	homogametic	heterogametic	Drosophilidae	Diptera	Insecta	XX, XY	(Navara 2018)
Teleopsis dalmanni	homogametic	heterogametic	Diopsidae	Diptera	Insecta	XX, XY	(Warren *et al.* 2010)
Aedes geniculatus	homogametic	heterogametic	Culicidae	Diptera	Insecta	mm, Mm	(Koskinioti *et al.* 2021)
Aedes albopictus	homogametic	heterogametic	Culicidae	Diptera	Insecta	mm, Mm	(Craig & Hickey 1967)
Heliconius erato	heterogametic	homogametic	Nymphalidae	Lepidoptera	Insecta	ZW, ZZ	(Van Belleghem *et al.* 2018)
Bicyclus anynana	heterogametic	homogametic	Nymphalidae	Lepidoptera	Insecta	WZ, ZZ	(Van't Hof *et al.* 2008)
Pieris napi	heterogametic	homogametic	Pieridae	Lepidoptera	Insecta	ZW, ZZ	(Keehnen *et al.* 2018)
Stator limbatus	homogametic	heterogametic	Chrysomelidae	Coleoptera	Insecta	XX, XO	(Arnqvist *et al.* 2015)
Callosobruchus maculatus	homogametic	heterogametic	Chrysomelidae	Coleoptera	Insecta	XX, XO	(Savalli & Fox 1998)
Phaedon cochleariae	homogametic	heterogametic	Chrysomelidae	Coleoptera	Insecta	XX, XO	(Arnqvist *et al.* 2015)
Euscepes postfasciatus	homogametic	heterogametic	Curculionidae	Coleoptera	Insecta	XX, XO	(Suomalainen 1940)
Ips pini	homogametic	heterogametic	Curculionidae	Coleoptera	Insecta	XX, XO	(Sargent & Reid, 1999)
Tenebrio molitor	homogametic	heterogametic	Tenebrionidae	Coleoptera	Insecta	XX, XO	(Arnqvist *et al.* 2015)
Tribolium castaneum	homogametic	heterogametic	Tenebrionidae	Coleoptera	Insecta	XX, XO	(Arnqvist *et al.* 2015)
Nicrophorus vespilloides	homogametic	heterogametic	Silphidae	Coleoptera	Insecta	XX, XO	(Angus & Diaz, 1991)
Narnia femorata	homogametic	heterogametic	Coreidae	Hemiptera	Insecta	XX, XO	(Dutt 1959)
Teleogryllus oceanicus	homogametic	heterogametic	Gryllidae	Orthoptera	Insecta	XX, XO	(Moran *et al.* 2018)
Teleogryllus commodus	homogametic	heterogametic	Gryllidae	Orthoptera	Insecta	XX, XO	(Moran *et al.* 2018)
Gryllodes sigillatus	homogametic	heterogametic	Gryllidae	Orthoptera	Insecta	XX, XY	(Rao & Arora 1979)
Forficula auricularia	homogametic	heterogametic	Forficulidae	Dermaptera	Insecta	XX, XY	(Rankin & Palmer 2009)
Stegodyphus lineatus	homogametic	heterogametic	Eresidae	Araneae	Arachnida	$X_1X_1X_2X_2$, X_1X_20	(Cordellier *et al.* 2020)

Rhizoglyphus robini	homogametic	heterogametic	Acaridae	Sarcoptiformes	Arachnida	XX, XO	(Radwan 1995)
Caenorhabditis remanei	homogametic	heterogametic	Rhabditidae	Rhabditida	Secernentea	XX, XO	(Baird & Seibert 2013)
Physa acuta	NA	NA	Physidae	Hygrophila	Gastropoda		
Poeciliopsis occidentalis	heterogametic	homogametic	Poeciliidae	Cyprinodontiformes	Actinopterygii	XY, XX	(Yamamoto & Kajishima 1968)
Gambusia holbrooki	homogametic	heterogametic	Poeciliidae	Cyprinodontiformes	Actinopterygii	XX, XY	(Senior *et al.* 2013)
Heterandria formosa	NA	NA	Poeciliidae	Cyprinodontiformes	Actinopterygii		
Poecilia reticulata	homogametic	heterogametic	Poeciliidae	Cyprinodontiformes	Actinopterygii	XX, XY	(Tomohisa *et al.* 2005)
Pelvicachromis taeniatus	homogametic	heterogametic	Cichlidae	Cichliformes	Actinopterygii	XX, XY	(Pandian 2011)
Oreochromis niloticus	homogametic	heterogametic	Cichlidae	Cichliformes	Actinopterygii	XX, XY	(Lee *et al.* 2003)
Danio rerio	NA	NA	Cyprinidae	Cypriniformes	Teleostei		
Microtus oeconomus	homogametic	heterogametic	Cricetidae	Rodentia	Mammalia	XX, XY	(Raman & Das 1991)
Microtus ochrogaster	homogametic	heterogametic	Cricetidae	Rodentia	Mammalia	XX, XY	(Borodin *et al.* 2012)
Peromyscus leucopus	homogametic	heterogametic	Cricetidae	Rodentia	Mammalia	XX, XY	(Waters *et al.* 2007)
Peromyscus polionotus	homogametic	heterogametic	Cricetidae	Rodentia	Mammalia	XX, XY	(Waters *et al.* 2007)
Parotomys littledalei	homogametic	heterogametic	Muridae	Rodentia	Mammalia	XX, XY	(Waters *et al.* 2007)
Mus musculus domesticus	homogametic	heterogametic	Muridae	Rodentia	Mammalia	XX, XY	(Raman & Das 1991)
Serinus canaria	heterogametic	homogametic	Fringillidae	Passeriformes	Aves	ZW, ZZ	(Doosti *et al.* 2009)

References

Arnqvist, G., Sayadi, A., Immonen, E., Hotzy, C., Rankin, D., Tuda, M. *et al.* (2015). Genome size correlates with reproductive fitness in seed beetles. *Proceedings of the Royal Society B: Biological Sciences*, 282, 20151421.

Baird, S.E. & Seibert, S.R. (2013). Reproductive isolation in the Elegans-Group of *Caenorhabditis*. *Natural Science*, 5, 18.

Borodin, P.M., Basheva, E.A., Torgasheva, A.A., Dashkevich, O.A., Golenishchev, F.N., Kartavtseva, I.V. *et al.* (2012). Multiple independent evolutionary losses of XY pairing at meiosis in the grey voles. *Chromosome Research*, 20, 259-268.

Cordellier, M., Schneider, J.M., Uhl, G. & Posnien, N. (2020). Sex differences in spiders: from phenotype to genomics. *Development Genes and Evolution*, 230, 155-172.

Craig, G.B., Jr. & Hickey, W.A. (1967). Current status of the formal genetics of *Aedes aegypti*. *Bulletin of The World Health Organization*, 36, 559-562.

Doosti, A., Fathpour, H. & Moshkelani, S. (2009). Sex identification in the Canary using DNA typing methods. *Bulgarian Journal of Veterinary Medicine*, 12.

Dutt, M. (1959). Cytology of three species of coreid bugs with special reference to multiple sex chromosome mechanism. *Genetica*, 29, 110-119.

Keehnen, N.L.P., Hill, J., Nylin, S. & Wheat, C.W. (2018). Microevolutionary selection dynamics acting on immune genes of the green-veined white butterfly, *Pieris napi*. *Molecular Ecology*, 27, 2807-2822.

Koskinioti, P., Augustinos, A.A., Carvalho, D.O., Misbah-ul-Haq, M., Pillwax, G., de la Fuente, L.D. *et al.* (2021). Genetic sexing strains for the population suppression of the mosquito vector *Aedes aegypti*. *Philosophical Transactions of the Royal Society B: Biological Sciences*, 376, 20190808.

Lee, B.Y., Penman, D.J. & Kocher, T.D. (2003). Identification of a sex-determining region in Nile tilapia (*Oreochromis niloticus*) using bulked segregant analysis. *Animal Genetics*, 34, 379-383.

Moran, P.A., Pascoal, S., Cezard, T., Risse, J.E., Ritchie, M.G. & Bailey, N.W. (2018). Opposing patterns of intraspecific and interspecific differentiation in sex chromosomes and autosomes. *Molecular Ecology*, 27, 3905-3924.

Navara, K.J. (2018). The truth about Nemo's dad: Sex-changing behaviors in fishes. In: *Choosing Sexes*. Springer, pp. 183-212.

Pandian, T. (2011). *Sex determination in fish*. CRC press.

Radwan, J. (1995). Male morph determination in two species of acarid mites. *Heredity*, 74, 669-673.

Raman, R. & Das, P. (1991). Mammalian sex chromosomes III. Activity of pseudoautosomal steroid sulfatase enzyme during spermatogenesis in *Mus musculus*. *Somatic Cell and Molecular Genetics*, 17, 429-433.

Rankin, S.M. & Palmer, J.O. (2009). Chapter 70 - Dermaptera: (Earwigs). In: *Encyclopedia of Insects (Second Edition)* (eds. Resh, VH & Cardé, RT). Academic Press San Diego, pp. 259-261.

Rao, S. & Arora, P. (1979). Insect sex chromosomes. *Chromosoma*, 74, 241-252.

Savalli, U.M. & Fox, C.W. (1998). Genetic variation in paternal investment in a seed beetle. *Animal Behaviour*, 56, 953-961.

Senior, A.M., Krkosek, M. & Nakagawa, S. (2013). The practicality of Trojan sex chromosomes as a biological control: an agent based model of two highly invasive *Gambusia* species. *Biological Invasions*, 15, 1765-1782.

Suomalainen, E. (1940). Polyploidy in parthenogenetic Curculionidae. *Hereditas*, 26, 51-64.

Tomohisa, W., Minoru, Y., Masamichi, N. & Nobuhiko, T. (2005). Linkage Mapping of AFLP and Microsatellite DNA Markers with the Body Color- and Sex-Determining Loci in the Guppy *Poecilia reticulata*. *Zoological Science*, 22, 883-889.

Van Belleghem, S.M., Baquero, M., Papa, R., Salazar, C., McMillan, W.O., Counterman, B.A. *et al.* (2018). Patterns of Z chromosome divergence among *Heliconius* species highlight the importance of historical demography. *Molecular Ecology*, 27, 3852-3872.

Van't Hof, A.E., Marec, F., Saccheri, I.J., Brakefield, P.M. & Zwaan, B.J. (2008). Cytogenetic Characterization and AFLP-Based Genetic Linkage Mapping for the Butterfly Bicyclus anynana, Covering All 28 Karyotyped Chromosomes. *Plos One*, 3, e3882.

Warren, I.A., Fowler, K. & Smith, H. (2010). Germline transformation of the stalk-eyed fly, *Teleopsis dalmanni*. *BMC Molecular Biology*, 11, 86.

Waters, P.D., Wallis, M.C. & Graves, J.A.M. (2007). Mammalian sex—origin and evolution of the Y chromosome and SRY. In: *Seminars in Cell & Developmental Biology*. Elsevier, pp. 389-400.

Yamamoto, T.-O. & Kajishima, T. (1968). Sex hormone induction of sex reversal in the goldfish and evidence for male heterogamity. *Journal of Experimental Zoology*, 168, 215-221.

Section III. List of species included in the meta-analysis with their corresponding sexual size dimorphism.

Species	Family	Order	Class	Size Dimorphism	Trait measured	Source
Drosophila serrata	Drosophilidae	Diptera	Insecta	-0.0561	wing size	(Liefting *et al.* 2009)
Drosophila bunnanda	Drosophilidae	Diptera	Insecta			
Drosophila birchii	Drosophilidae	Diptera	Insecta	0.0654	wing size	(Kellermann *et al.* 2007)
Drosophila simulans	Drosophilidae	Diptera	Insecta	-0.1088	thorax length	(Pitnick *et al.* 1995)
Drosophila melanogaster	Drosophilidae	Diptera	Insecta	-0.1178	thorax length	(Pitnick *et al.* 1995)
Drosophila bipectinata	Drosophilidae	Diptera	Insecta	-0.2746	wing size	(Imasheva *et al.* 1999)
Drosophila pseudoananassae	Drosophilidae	Diptera	Insecta			
Drosophila repleta	Drosophilidae	Diptera	Insecta			
Drosophila sulfurigaster	Drosophilidae	Diptera	Insecta	-0.0135	wing length	(Neethu & Harini 2014)
Teleopsis dalmanni	Diopsidae	Diptera	Insecta	0.0342	thorax length	(Prokop *et al.* 2010)
Aedes geniculatus	Culicidae	Diptera	Insecta	-0.5251	body mass	(Bradshaw & Holzapfel 1992)
Aedes albopictus	Culicidae	Diptera	Insecta	-0.1867	wing size	(Sánchez *et al.* 2017)
Heliconius erato	Nymphalidae	Lepidoptera	Insecta	-0.0028	hind wing length	(Klein & de Araújo 2013)
Bicyclus anynana	Nymphalidae	Lepidoptera	Insecta	-0.2343	pupal mass	(Dierks *et al.* 2012)
Pieris napi	Pieridae	Lepidoptera	Insecta	0.0246		(Stjernholm & Karlsson 2000)
Stator limbatus	Chrysomelidae	Coleoptera	Insecta	0.0350	body mass	(Stillwell & Fox 2009)
Callosobruchus maculatus	Chrysomelidae	Coleoptera	Insecta	-0.0478	elytron length	(Colgoni & Vamosi 2006)
Phaedon cochleariae	Chrysomelidae	Coleoptera	Insecta	-0.4523	body mass	(Müller *et al.* 2016)
Euscepes postfasciatus	Curculionidae	Coleoptera	Insecta	-0.0175	elytra length	(Kumano *et al.* 2010)
Ips pini	Curculionidae	Coleoptera	Insecta	0.0118	body size	(Foelker & Hofstetter 2014)
Tenebrio molitor	Tenebrionidae	Coleoptera	Insecta	0.0268	body mass	(Rantala *et al.* 2011)
Tribolium castaneum	Tenebrionidae	Coleoptera	Insecta	-0.0196	protonum width	(Conner & Via 1992)
Nicrophorus vespilloides	Silphidae	Coleoptera	Insecta	-0.0253	elytra length	(Otronen 1988)
Narnia femorata	Coreidae	Hemiptera	Insecta	-0.0824	protonum width	(Allen & Miller 2017)
Teleogryllus oceanicus	Gryllidae	Orthoptera	Insecta	0.0507	protonum width	(Nystrand *et al.* 2011)
Teleogryllus commodus	Gryllidae	Orthoptera	Insecta	0.0697	body mass	(Drayton *et al.* 2011)
Gryllodes sigillatus	Gryllidae	Orthoptera	Insecta	-0.0673	femur length	(Galicia *et al.* 2014)
Forficula auricularia	Forficulidae	Dermaptera	Insecta	0.2136	elytra length	(Radesäter & Halldórsdóttir 1993)
Stegodyphus lineatus	Eresidae	Araneae	Arachnida	-0.0866	body size	(Schneider 1997)

Rhizoglyphus robini	Acaridae	Sarcoptiformes	Arachnida	-0.2016	body size	(Deere *et al.* 2015)
Caenorhabditis remanei	Rhabditidae	Rhabditida	Secernentea	NA		
Physa acuta	Physidae	Hygrophila	Gastropoda	NA		
Poeciliopsis occidentalis	Poeciliidae	Cyprinodontiformes	Actinopterygii	-0.3331	body size	(Constanz 1975)
Gambusia holbrooki	Poeciliidae	Cyprinodontiformes	Actinopterygii	-0.3506	body size	(Bisazza & Pilastro 1997)
Heterandria formosa	Poeciliidae	Cyprinodontiformes	Actinopterygii	-0.2345	body size	(Bisazza & Pilastro 1997)
Poecilia reticulata	Poeciliidae	Cyprinodontiformes	Actinopterygii	-0.3523	body size	(Bisazza & Pilastro 1997)
Pelvicachromis taeniatus	Cichlidae	Cichliformes	Actinopterygii	0.3538	body size	(Meuthen *et al.* 2018)
Oreochromis niloticus	Cichlidae	Cichliformes	Actinopterygii	0.0657	body size	(Nguyen *et al.* 2007)
Danio rerio	Cyprinidae	Cypriniformes	Teleostei	-0.0843	body size	(Uusi-Heikkilä *et al.* 2012)
Microtus oeconomus	Cricetidae	Rodentia	Mammalia	0.0180	body mass	(Tast 1972)
Microtus ochrogaster	Cricetidae	Rodentia	Mammalia	0.0732	body mass	(Dewsbury *et al.* 1980)
Peromyscus leucopus	Cricetidae	Rodentia	Mammalia	0.0723	body mass	(Dewsbury *et al.* 1980)
Peromyscus polionotus	Cricetidae	Rodentia	Mammalia	-0.0192	body mass	(Dewsbury *et al.* 1980)
Parotomys littledalei	Muridae	Rodentia	Mammalia	0.1955	body mass	(Pillay 2002)
Mus musculus domesticus	Muridae	Rodentia	Mammalia	0.1405	body mass	(Dewsbury *et al.* 1980)
Serinus canaria	Fringillidae	Passeriformes	Aves	0.0223	tarsus length	(de Boer *et al.* 2016)

References

Allen, P.E. & Miller, C.W. (2017). Novel host plant leads to the loss of sexual dimorphism in a sexually selected male weapon. *Proceedings of the Royal Society B: Biological Sciences*, 284, 20171269.

Bisazza, A. & Pilastro, A. (1997). Small male mating advantage and reversed size dimorphism in poeciliid fishes. *Journal of Fish Biology*, 50, 397-406.

Bradshaw, M.E. & Holzapfel, C.M. (1992). Resource limitation, habitat segregation, and species interactions of british tree-hole mosquitoes in nature. *Oecologia*, 90, 227-237.

Colgoni, A. & Vamosi, S.M. (2006). Sexual dimorphism and allometry in two seed beetles (Coleoptera: Bruchidae). *Entomological Science*, 9, 171-179.

Conner, J. & Via, S. (1992). Natural selection on body size in *Tribolium*: possible genetic constraints on adaptive evolution. *Heredity*, 69, 73-83.

Constanz, G.D. (1975). Behavioral Ecology of Mating in the Male Gila Topminnow, *Poecilipsis occidentalis* (Cyprinodontiformes: Poeciliidae). *Ecology*, 56, 966-973.

de Boer, R.A., Eens, M. & Müller, W. (2016). A loss of heterozygosity, a loss in competition? The effects of inbreeding, pre- and postnatal conditions on nestling development. *Ecology and Evolution*, 6, 7921-7930.

Deere, J.A., Coulson, T. & Smallegange, I.M. (2015). Life History Consequences of the Facultative Expression of a Dispersal Life Stage in the Phoretic Bulb Mite (*Rhizoglyphus robini*). *Plos One*, 10, e0136872.

Dewsbury, D.A., Baumgardner, D.J., Evans, R.L. & Webster, D.G. (1980). Sexual Dimorphism for Body Mass in 13 Taxa of Muroid Rodents under Laboratory Conditions. *Journal of Mammalogy*, 61, 146-149.

Dierks, A., Baumann, B. & Fischer, K. (2012). Response to selection on cold tolerance is constrained by inbreeding. *Evolution*, 66, 2384-2398.

Drayton, J.M., Milner, R.N.C., Hall, M.D. & Jennions, M.D. (2011). Inbreeding and courtship calling in the cricket *Teleogryllus commodus*. *Journal of Evolutionary Biology*, 24, 47-58.

Foelker, C.J. & Hofstetter, R.W. (2014). Heritability, Fecundity, and Sexual Size Dimorphism in Four Species of Bark Beetles (Coleoptera: Curculionidae: Scolytinae). *Annals of the Entomological Society of America*, 107, 143-151.

Galicia, A., Cueva del Castillo, R. & Contreras-Garduño, J. (2014). Is Sexual Dimorphism in the Immune Response of *Gryllodes sigillatus* Related to the Quality of Diet? *ISRN Evolutionary Biology*, 2014, 329736.

Imasheva, A.G., Bosenko, D.V. & Bubli, O.A. (1999). Variation in morphological traits of *Drosophila melanogaster* (fruit fly) under nutritional stress. *Heredity*, 82, 187-192.

Kellermann, V.M., Hoffmann, A.A. & SgrÒ, C.M. (2007). Hsp90 inhibition and the expression of phenotypic variability in the rainforest species Drosophila birchii. *Biological Journal of the Linnean Society*, 92, 457-465.

Klein, A.L. & de Araújo, A.M. (2013). Sexual Size Dimorphism in the Color Pattern Elements of Two Mimetic *Heliconius* Butterflies. *Neotropical Entomology*, 42, 600-606.

Kumano, N., Kuriwada, T., Shiromoto, K., Haraguchi, D. & Kohama, T. (2010). Effect of body size and sex ratio on male alternative mating tactics of the West Indian sweetpotato weevil, *Euscepes postfasciatus*. *Entomologia Experimentalis et Applicata*, 135, 154-161.

Liefting, M., Hoffmann, A.A. & Ellers, J. (2009). Plasticity versus environmental canalization: population differences in thermal responses along a latitudinal gradient in *Drosophila serrata*. *Evolution*, 63, 1954-1963.

Meuthen, D., Baldauf, S.A., Bakker, T.C.M. & Thünken, T. (2018). Neglected Patterns of Variation in Phenotypic Plasticity: Age- and Sex-Specific Antipredator Plasticity in a Cichlid Fish. *The American Naturalist*, 191, 475-490.

Müller, T., Küll, C.L. & Müller, C. (2016). Effects of larval versus adult density conditions on reproduction and behavior of a leaf beetle. *Behavioral Ecology and Sociobiology*, 70, 2081-2091.

Neethu, B. & Harini, B. (2014). Implications of macronutrient supplements causes stress on life history traits in immigrans species group of *Drosophila sulfurigaster sulfurigaster*.

Nguyen, N.H., Khaw, H.L., Ponzoni, R.W., Hamzah, A. & Kamaruzzaman, N. (2007). Can sexual dimorphism and body shape be altered in Nile tilapia (*Oreochromis niloticus*) by genetic means? *Aquaculture*, 272, S38-S46.

Nystrand, M., Dowling, D.K. & Simmons, L.W. (2011). Complex Genotype by Environment interactions and changing genetic architectures across thermal environments in the Australian field cricket, *Teleogryllus oceanicus*. *BMC Evolutionary Biology*, 11, 222.

Otronen, M. (1988). The effect of body size on the outcome of fights in burying beetles (*Nicrophorus*). *Annales Zoologici Fennici*, 25, 191-201.

Pillay, N. (2002). Inbreeding in Littledale's whistling rat *Parotomys littledalei*. *J Exp Zool*, 293, 171-178.

Pitnick, S., Markow, T.A. & Spicer, G.S. (1995). Delayed male maturity is a cost of producing large sperm in *Drosophila*. *Proceedings of the National Academy of Sciences of the United States of America*, 92, 10614-10618.

Prokop, Z.M., Le, J.E., Bana, P.K., Koteja, P. & Radwan, J. (2010). Low inbreeding depression in a sexual trait in the stalk-eyed fly *Teleopsis dalmanni*. *Evolutionary Ecology*, 24, 827-837.

Radesäter, T. & Halldórsdóttir, H. (1993). Fluctuating asymmetry and forceps size in earwigs, *Forficula auricularia*. *Animal Behaviour*, 45, 626-628.

Rantala, M.J., Viitaniemi, H. & Roff, D.A. (2011). Effects of inbreeding on potential and realized immune responses in *Tenebrio molitor*. *Parasitology*, 138, 906-912.

Sánchez, E., Castillo, D. & Liria, J. (2017). Pupal shape and size dimorphism in *Aedes albopictus* (Skuse, 1894) (Diptera: Culicidae). *Journal of Threatened Taxa*, 6, 10314–10319.

Schneider, J.M. (1997). Timing of maturation and the mating system of the spider,*Stegodyphus lineatus* (Eresidae): how important is body size? *Biological Journal of the Linnean Society*, 60, 517-525.

Stillwell, R.C. & Fox, C.W. (2009). Geographic variation in body size, sexual size dimorphism and fitness components of a seed beetle: local adaptation versus phenotypic plasticity. *Oikos*, 118, 703-712.

Stjernholm, F. & Karlsson, B. (2000). Nuptial gifts and the use of body resources for reproduction in the green–veined white butterfly *Pieris napi*. *Proceedings of the Royal Society of London. Series B: Biological Sciences*, 267, 807-811.

Tast, J. (1972). Annual variations in the weights of wintering root voles, *Microtus oeconomus*, in relation to their food conditions. *Annales Zoologici Fennici*, 9, 116-119.

Uusi-Heikkilä, S., Böckenhoff, L., Wolter, C. & Arlinghaus, R. (2012). Differential Allocation by Female Zebrafish (*Danio rerio*) to Different-Sized Males – An Example in a Fish Species Lacking Parental Care. *Plos One*, 7, e48317.
